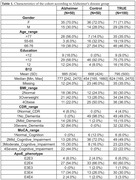# Role of the Gut Microbiome in Cognitive Health of Puerto Ricans: A Novel Perspective for Understanding Alzheimer’s Disease

**DOI:** 10.1002/alz.095694

**Published:** 2025-01-09

**Authors:** Carlos Alberto Herrero‐Rivera, Hiram Morales, Cecilia Michelle Soler‐Llompart, Ana Cecilia Sala‐Morales, Gerianne Olivieri‐Henry, Filipa Godoy‐Vitorino, Vanessa Sepulveda

**Affiliations:** ^1^ University of Puerto Rico, Medical Sciences Campus, School of Medicine, San Juan, PR USA; ^2^ University of Puerto Rico, Medical Sciences Campus, San Juan, PR USA; ^3^ University of Puerto Rico, Río Piedras Campus, San Juan, PR USA

## Abstract

**Background:**

Alzheimer’s disease (AD) comprise over 70% of dementia cases in the United States (US), characterized by progressive neurodegenerative symptoms. In the US and Puerto Rico (PR), AD is the sixth and fourth leading cause of death, respectively. The Gut‐Brain Axis is a bidirectional communication mechanism between the central nervous system and microbes that reside in the gastrointestinal tract. Research shows that neuroactive metabolites influence neurological responses and inflammatory pathways. Even though a correlation between gut microbiome and some neuropsychological diseases (e.g., Parkinson’s) has been described, its association with AD is understudied. This study compared the gut microbiome of Puerto Ricans with AD to healthy controls to help elucidate the role of gut microbiota as a possible contributing factor for the high prevalence in this population.

**Method:**

The study evaluated 100 participants: AD (n = 50) and controls (n = 50) with a medical history, physical exam, ApoE genotype, and cognition using the Montreal Cognitive Assessment (MoCA) and Clinical Dementia Rating Scale (CDR). Stool for genomic DNA extractions and microbiome characterization using 16S rRNA genes (V4 region) via Illumina MiSeq.

**Result:**

Microbial Alpha Diversity showed no significant difference between AD and controls. Microbial Beta Diversity showed a significant difference between groups (P‐value = 0.034). Phyla‐level taxa tendencies in AD showed a higher relative abundance of Actinobacteria, Verrucomicrobia, and Euryarchaeota. Taxonomic profiling also showed reduced levels of neuroprotective genera Faecalibacterium and Bacteroides, and increased levels of pro‐inflammatory opportunistic Eggerthella. Regarding CDR testing of AD groups, there were significant differences in alpha diversity among normal CDR with mild and severe dementia. The microbial richness of controls declined with their cognitive status based on MoCA. One variant of ApoE4 was present in 92% of AD patients (46/50) and 94% in controls (47/50). Only one subject had E3E3 phenotype while 30.4% E3E4, 2.9% E4E4, and 5.9% E2E3.

**Conclusion:**

This study, the first of its kind in PR, suggests that microbial diversity and richness are associated with cognitive status. Furthermore, it showed possible bacterial dysbiosis in AD patients compared to controls which paves the way for identifying microbial biomarkers and modulation‐based interventions.